# Intrahousehold power inequalities and cooperation: Unpacking household responses to nutrition‐sensitive agriculture interventions in rural India

**DOI:** 10.1111/mcn.13503

**Published:** 2023-03-20

**Authors:** Helen Harris‐Fry, Audrey Prost, Emma Beaumont, Emily Fivian, Satyanarayan Mohanty, Manoj Parida, Ronali Pradhan, Satyapriya Sahu, Shibanath Padhan, Naba K. Mishra, Shibanand Rath, Suchitra Rath, Peggy Koniz‐Booher, Elizabeth Allen, Suneetha Kadiyala

**Affiliations:** ^1^ Faculty of Epidemiology and Population Health London School of Hygiene & Tropical Medicine London UK; ^2^ Institute for Global Health University College London London UK; ^3^ Digital Green New Delhi India; ^4^ DCOR (Development Corner) Consulting Pvt. Ltd. Bhubaneswar India; ^5^ Voluntary Association for Rural Reconstruction and Appropriate Technology Kendrapara India; ^6^ Ekjut Chakradharpur Jharkhand India; ^7^ JSI Research & Training Institute Arlington Virginia USA

**Keywords:** cooperation, diets, household behaviour, India, mixed methods, nutrition‐sensitive agriculture, power

## Abstract

Nutrition‐sensitive agriculture (NSA) interventions offer a means to improve the dietary quality of rural, undernourished populations. Their effectiveness could be further increased by understanding how household dynamics enable or inhibit the uptake of NSA behaviours. We used a convergent parallel mixed‐methods design to describe the links between household dynamics—specifically intrahousehold power inequalities and intrahousehold cooperation—and dietary quality and to explore whether household dynamics mediated or modified the effects of NSA interventions tested in a cluster‐randomized trial, Upscaling Participatory Action and Videos for Agriculture and Nutrition (UPAVAN). We use quantitative data from cross‐sectional surveys in 148 village clusters at UPAVAN's baseline and 32 months afterwards (endline), and qualitative data from family case studies and focus group discussions with intervention participants and facilitators. We found that households cooperated to grow and buy nutritious foods, and gendered power inequalities were associated with women's dietary quality, but cooperation and women's use of power was inhibited by several interlinked factors. UPAVAN interventions were more successful in more supportive, cooperative households, and in some cases, the interventions increased women's decision‐making power. However, women's decisions to enter into negotiations with family members depended on whether women deemed the practices promoted by UPAVAN interventions to be feasible, as well as women's confidence and previous cultivation success. We conclude that interventions may be more effective if they can elicit cooperation from the whole household. This will require a move towards more family‐centric intervention models that empower women while involving other family members and accounting for the varied ways that families cooperate and negotiate.

## Key messages

- Household cooperation and intrahousehold power inequalities may influence children's and mothers' dietary diversity in rural Odisha
- These factors partly determine the effects of nutrition‐sensitive agriculture (NSA) interventions tested in Upscaling Participatory Action and Videos for Agriculture and Nutrition, indicating that NSA interventions may be more effective if they include multiple family members.
- There is wide heterogeneity in household dynamics and the constraints that families face. Further research is needed to identify how interventions can respond to this heterogeneity so that NSA interventions are effective in an inclusive and empowering way.

## Graphical abstract

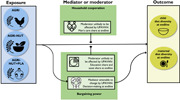

## 1 INTRODUCTION

In South Asia, the burden of undernutrition is the highest in the world: a third of children and a quarter of women are chronically undernourished (Global Nutrition Report, 2020; Victora et al., 2021). To address this, we not only need effective interventions that address the direct causes of undernutrition (termed ‘nutrition‐specific’ interventions), but also effective ‘nutrition‐sensitive’ interventions that address root causes of undernutrition, such as low agricultural productivity, food insecurity and gender‐based inequities (Bhutta et al., 2013; Kadiyala et al., 2014). Nutrition‐sensitive agriculture (NSA) interventions offer a means to do this and can improve dietary quality in several settings (Margolies et al., 2022). However, they are typically less cost‐effective than nutrition‐specific interventions (Webb et al., 2021). To enhance the impact and thus cost‐effectiveness of NSA programmes, a better understanding is needed of how they work, who they benefit most, and how we can maximize effectiveness for the whole population.

Two factors that may mediate and/or modify NSA intervention impacts are intrahousehold power inequalities and intrahousehold cooperation. So far, most impact evaluations of NSA interventions have focused on power inequalities, particularly gendered power inequalities. In India, gendered power inequalities can be observed across multiple domains of life, including women's lower social mobility, political representation, access to education, participation and wage rates in the labour market and control over assets such as land (Mahajan, 2019; Rammohan & Vu, 2018; Santos et al., 2014). Within the home, gendered power inequalities persist: women often have higher workloads, and limited authority in decision‐making (Aakesson et al., 2017; Nichols, 2016; Srinivasan et al., 2020).

Recognizing this, it is often assumed that reducing gendered power inequalities will result in larger shares of the household budget being spent on food and health care. However, studies from across the world (Harris‐Fry, Nur, et al., 2020) and within India (Chaturvedi et al., 2016; Gaiha & Kulkarni, 2005; Lancaster et al., 2006) have shown impressively heterogeneous effects of gendered power inequalities on food expenditures and nutrition outcomes for women and children. Consistent with this, a review of NSA interventions has shown that the extent to which gendered power inequalities mediate intervention effectiveness is highly variable (Sharma et al., 2021). Evidence of moderation by gendered power inequalities has also been documented, although to a lesser extent (Gilligan et al., 2020).

There is now a growing recognition of the importance of cooperation among family members, and this is increasingly being factored into the design of nutrition interventions (Morrison et al., 2021; Thuita et al., 2021). Cooperation may occur in several ways, for example, by sharing risk and information, sharing tasks and gains from agricultural production and sharing the responsibilities of raising children. Some studies have highlighted men's role in supporting their wife's access to antenatal and post‐natal care (Barua et al., 2004; Morrison et al., 2021), and their children's access to an adequate diet (Han et al., 2019; Nyqvist & Jayachandran, 2017), while a growing literature has highlighted the role of grandmothers in improving nutrition outcomes (Aubel, 2012; Concha & Jovchelovitch, 2021; Negin et al., 2016).

Although it is easy to imagine that household cooperation will determine NSA intervention effectiveness, evidence on the role of household cooperation in determining NSA intervention impacts is scant (Sharma et al., 2021). This paper seeks to address these gaps by (1) examining the role of intrahousehold power inequalities and intrahousehold cooperation in determining the dietary quality of mothers and children in rural Odisha, India and (2) unpacking the role that these household dynamics play in determining the effectiveness of three NSA interventions tested in rural Odisha. The interventions were tested in a cluster‐randomized controlled trial, ‘UPAVAN’ (Upscaling Participatory Action and Videos for Agriculture and Nutrition). The main impact evaluation showed that each intervention increased maternal and/or child minimum dietary diversity (Kadiyala et al., 2021). The main process evaluation found that land and water constraints, gender norms, and lack of support from family members prevented some households from changing their cultivation and dietary practices (Prost et al., 2022). We build on this work using a mixed‐methods approach to examine whether gendered power inequalities and intrahousehold cooperation are associated with the dietary quality of mothers and children in rural India, and then test whether these household dynamics moderated or mediated the effects of UPAVAN interventions.

## 2 METHODS

### 2.1 UPAVAN overview

UPAVAN was a four‐arm cluster‐randomized controlled trial that was implemented in 148 clusters (villages and surrounding hamlets), from four administrative blocks in Keonjhar district, Odisha. Keonjhar is a land‐locked, heavily forested district with a growing population of around 1.8 million people. There are low levels of absolute landlessness, but 36% of households in rural Odisha live below the poverty line (Government of Odisha, 2021). Many people belong to Scheduled Tribes (45%)—historically disadvantaged groups that are prioritized in some government schemes (IFPRI, 2016; Census of India, 2011). Poverty, small landholdings, food insecurity and undernutrition are all more common in Tribal groups (Das & Bose, 2015; K. Kumar et al., 2005; Mehta, 2011).

Cropping practices in Odisha mostly rely on rainfed agriculture, so crop and livestock productivity is low, often unprofitable and threatened by cyclones, floods and drought (Arora & Birwal, 2017; Singh, 2013). Many diversify incomes through migration, mining, trade, public transfers, daily wage labour for public work schemes and foraging (Rew & Rew, 2003; Savath et al., 2014). Dietary quality is low. The baseline report showed that around 80% of women and children did not access an adequately diverse diet, and 44% of children and 29% of women in Keonjhar were underweight (NFHS‐4, 2016).

Seeking to find effective ways to improve maternal and child dietary diversity and nutritional status through agricultural interventions, the UPAVAN trial developed and tested three NSA interventions, each compared to a control arm of standard government services (Kadiyala et al., 2021). The interventions were delivered at the cluster level (with 37 clusters per arm) and all women living in intervention clusters were eligible to participate. Impacts were evaluated in children aged 0–23 months, their mothers, and their households.

The core components of all three interventions were: (i) women's group meetings (two meetings/month/group, over a 32‐month period), and (ii) follow‐up home visits to group participants if they were pregnant women or mothers of children aged 0–23 months. The group meetings were primarily run through women's Self‐Help Groups (SHGs)—a platform for savings and credit—but with an added effort to increase group coverage and participation. We chose the SHG platform because it has been shown to increase gender equity in empowerment indicators (N. Kumar et al., 2021), there is a large body of evidence showing SHGs in India can improve women's and children's health (Desai et al., 2020), and because of the policy impetus to SHGs and the associated potential for scale‐up. Furthermore, this platform would enable us to include women farmers who own small plots of land, and who are traditionally excluded from agricultural extension (Anderson, 2006; Swanson, 2008). Where groups did not exist new ones were formed, and—since SHG members are often older women (including mothers‐in‐law and/or grandmothers)—group facilitators expanded participation by inviting other members, particularly pregnant women and mothers of children aged 0–23 months.

The key differences between the arms were content in the meetings:

1. In the first intervention (‘AGRI’), facilitators screened locally made videos on NSA practices using low‐cost projectors and fostered discussion around key messages. NSA videos covered topics on increasing food production or agricultural income, reducing agricultural workload, and increasing women's decision‐making. Videos on women's decision‐making included demonstrations of family budgeting and crop planning exercises.
2. In the second intervention (‘AGRI‐NUT’), facilitators showed videos on both NSA (half of those in AGRI) and nutrition‐specific practices. Nutrition‐specific videos covered topics on maternal and child diets.
3. In the third intervention (‘AGRI‐NUT+PLA’), meetings showed videos on NSA (half of those in AGRI) and followed a nutrition‐specific participatory learning and action (PLA) approach. The PLA meeting cycle involved a mix of nutrition‐specific videos and participatory meetings without videos and was constructed as a cycle of four phases. In the first phase, groups identify and prioritize nutrition problems and learn together. Second, they prioritize solutions and strategies to collectively address these problems. In the third phase, they act together to implement these strategies, and in the fourth phase, the groups evaluate together and decide upon their next steps.

Pregnant women and mothers of children aged 0–23 who attended the groups received a follow‐up visit in their homes after each meeting. These visits aimed to build more rapport with the participants, check if participants could recall and/or had adopted any practices promoted in the last meeting, strengthen links to government frontline workers when appropriate and encourage attendance at the next meeting. Some facilitators also took this opportunity to interact with other household members who could enable or inhibit the uptake of promoted practices (Prost et al., 2022).

Videographers and group facilitators were salaried, trained local staff employed by the implementing organization. The calendar of videos and PLA meeting manual was informed by our theory of change (Supporting Information: Figure 1), the team's knowledge, published literature, formative qualitative research and community feedback. The videos themselves featured many family members—including supportive husbands and mothers‐in‐law—and wider community members such as frontline health and agricultural workers.

Elsewhere we report on the formative research (Aakesson et al., 2017; Kadiyala et al., 2016), intervention development process (Harris‐Fry, O'Hearn, et al., 2020), protocol (Kadiyala et al., 2018), impact evaluation (Kadiyala et al., 2021), main process evaluation (Prost et al. 2022) and cost‐effectiveness evaluation (Haghparast‐Bidgoli et al., 2022).

### 2.2 Quantitative data collection

Our mixed‐methods approach uses data from the UPAVAN trial. We use cross‐sectional surveys at baseline (November 2016 to January 2017) and endline (November 2019 to January 2020), plus secondary analysis of qualitative data from a process evaluation conducted over two phases (March to April 2018 and March to April 2019). Our analysis used a convergent parallel mixed‐methods design, which involved collecting and analyzing quantitative and qualitative data separately and then integrating the findings together in our overall interpretation (Creswell, 2014).

For baseline and endline surveys, we selected a random sample of households with a child aged 0–23 months and a female primary caregiver aged 15–49 years. We aimed to sample 32 households per cluster in all 148 clusters, giving a target sample of 4736 households.

A trained data collection team administered a pretested questionnaire on a range of indicators. Variables used in this paper are given in Table 1.

**Table 1:** Variable definitions.

Construct	Indicator	Indicator definition
Women's relative power	Women's asset share	Women's asset count/household asset count. Women's asset counts have values of 0, 0.5 or 1 (for none, joint or sole ownership). In households where households have zero assets, the share is 0.5. We only count large assets that would not have changed due to UPAVAN interventions, which were: agricultural land, nonagricultural land, house, large livestock, small livestock, mechanized farm equipment, nonmechanized farm equipment, business equipment, high‐cost durables, phone and jewellery.
	Women's education share	Women/(women + spouse) years of completed formal education. In households where men and women have no education, the share is 0.5.
	Women's decision‐making	The number of decisions woman is typically involved in, out of 7. For each decision, women have a score of 0, 0.5 or 1 (for none, some or all/most input). Decisions relate to food cropping, cash cropping, animal husbandry, nonfarm business, minor daily food expenditures, accessing markets and seeking health care.
Household cooperation	Household cooperation, indicated as men's care share	Men's care share = men/(men + women) hours spent on childcare in the previous 24 h. We measured men's childcare in a random selection of half the sample, and this excludes women‐only households and cases where the male respondent was not the husband.
Child diets	Child dietary diversity score	Count out of seven food groups. Food groups are grains; roots and tubers; legumes and nuts; dairy products; flesh foods; eggs; vitamin A‐rich fruits and vegetables and other fruits and vegetables) (WHO, 2010). Measured for children aged 6–23 months only.
Women's diets	Maternal dietary diversity score	Count out of 10 food groups. Food groups are: starchy staples; bean, peas and pulses; nuts and seeds; eggs; meat and fish; dairy; dark green leafy vegetables; other vitamin A‐rich fruits and vegetables, other fruits, other vegetables) (FAO, 2014).
Covariates	Household assets	Count out of 11 household assets.
	Total education	Total number of years of education, summed for father and mother.
	Caste	Three categories: Scheduled Caste, Scheduled Tribe and other castes including other backward castes.
	Household dependency ratio	Number of children/adults.
	Land size	Binary indicator (<2.5 vs. ≥2.5 ac).
	Maternal age	Age in years.
	Child age	Age in months.

Abbreviation: UPAVAN, Upscaling Participatory Action and Videos for Agriculture and Nutrition.

To indicate intrahousehold power inequalities, we used the following proxy indicators of women's relative power: women's share of household assets, women's share of education and a count of decisions that women make concerning domestic and farm management (Chiappori et al., 2018; Doss, 2013; Kabeer, 1999; Malapit et al., 2015; Quisumbing & Maluccio, 2003).

Following Lewbel and Pendakur (2022), we indicate intrahousehold cooperation using the father's share of time spent on childcare (hereafter ‘men's care share’). Sharing the responsibilities of childcare constitutes a key way in which parents cooperate since it requires a large time and financial investment, and children usually constitute an important part of family life (Gobbi, 2018). Men's time use was measured in 50% of households, so any analyses with this indicator are restricted to this subsample.

### 2.3 Quantitative data analysis

We first use baseline data to describe associations of relative power and household cooperation with diet diversity score, excluding women‐only households, unmarried women, and men who are not spouses. We report cross‐sectional associations, using multivariable mixed‐effect linear regression with a random effect for the study cluster. Adjusted models included prehypothesized confounders (covariates in Table 1). Where the maternal dietary diversity score is the outcome we adjust for maternal age, and where the child dietary diversity score is the outcome, we adjust for the child's age. For associations between cooperation and diets, we also include the total time spent on childcare.

**Figure 1:**
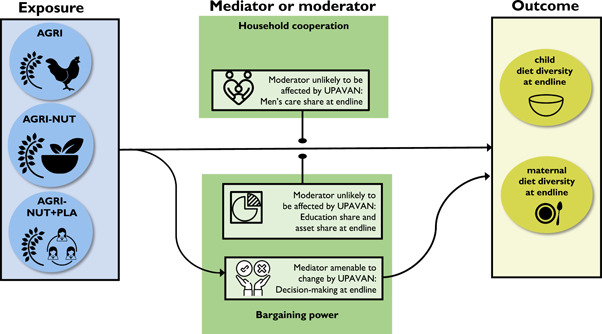
Analyses used to explore the effects of UPAVAN interventions on dietary diversity. Arrows with circular ends indicate moderation; arrows with triangular points report impact paths. UPAVAN, Upscaling Participatory Action and Videos for Agriculture and Nutrition.

Next, we use data from all arms at baseline and endline in longitudinal analyses to test whether household cooperation or power inequalities moderate or mediate the effects of the UPAVAN interventions on dietary diversity score (Figure 1). To estimate the effects of the interventions on dietary diversity and possible mediators, we compare the outcomes in each intervention arm with the control arm at endline, adjusting for cluster‐level baseline measures of the outcome. All longitudinal analyses are by intention‐to‐treat and all models use multivariable mixed‐effect linear regression with a random effect for the study cluster.

#### 2.3.1 Moderation

To investigate whether intrahousehold power inequalities and/or household cooperation moderate the effect of the UPAVAN interventions on dietary diversity (arrows with circular ends in Figure 1), we use indicators of gendered power inequalities (asset share, education share) and cooperation (care share) that we did not explicitly aim to change in UPAVAN and hypothesized would not be affected by the interventions. Although moderators were measured at endline, we assume they proxy the level of the moderator at baseline. To check this, we test whether each hypothesized moderator differs by arm at endline. For those that do not differ, we report moderation of intervention effects by high (top 50%) versus low (bottom 50%) levels of the hypothesized moderator. We test for evidence of moderation by fitting an interaction term between moderator and exposure. We report the association between exposure and outcome at each level of the moderator and *p* value from a Wald test for the interaction terms.

#### 2.3.2 Mediation

We explore whether UPAVAN interventions were mediated by changes in one dimension of women's power that could (and we intended to) change through UPAVAN: women's decision‐making (arrows with triangular points in Figure 1).

To test for evidence of mediation, we follow the Baron and Kenny (1986) approach, by reporting whether all three of the following conditions are met:

1. The intervention affects the mediator (decision‐making).
2. The intervention affects the outcome (diets).
3. The mediator (decision‐making) affects the outcome (diets), controlling for intervention.

All statistical analyses were done using Stata (version 17).

### 2.4 Qualitative data collection

To describe how household cooperation or power inequalities might influence women's diets in more detail, we use qualitative data from UPAVAN's process evaluation. We carried out 17 focus group discussions with SHGs (5–6 per intervention arm), or a total of 181 group members. These discussions explored participation in SHG meetings, the effects of interventions on SHG members' own or others' diets, changes to cultivation and enablers and barriers to changes. We also conducted three focus group discussions (one per arm) with 32 intervention facilitators and supervisors to understand community responses to interventions. Finally, we compiled 32 family case studies using individual interviews with pregnant women, mothers of children under two, and their husbands, fathers and mothers‐in‐law (8 in AGRI, 12 in AGRI‐NUT and 12 in AGRI‐NUT‐PLA, totalling 91 semistructured interviews). Case studies focused on changes to diets in pregnancy or for young children, and barriers and enablers to the adoption of NSA practices.

Five researchers fluent in Odia and with between 2 and 8 years of experience in qualitative data collection collected the data over two phases (March–April 2018 and March–April 2019). The process evaluation team revised data collection strategies and tools after the first phase to include more focus group discussions with SHG members and to ensure that mothers included in case studies had attended some video or PLA meetings. We identified potential participants for focus groups and case studies by purposively selecting five clusters per intervention arm, stratified by the proportion of Scheduled Caste and/or Tribal families and distance from the nearest town. In each village, we approached mothers who had attended at least three meetings to take part in interviews, and SHG members were invited for focus group discussions. Topic guides are included in Prost et al. (2022).

### 2.5 Qualitative data analysis

We used recordings transcribed and translated audio recordings from Odia to English. Transcripts were the same as those used for the process evaluation (Prost et al., 2022). Characteristics of participants in the interviews used to construct case studies were included in Prost et al. (2022) as Supporting Information: Table 1. Focus group transcripts did not include individual age, caste/tribe or duration of SHG membership for each individual participant so as not to identify them. In the original process evaluation, we used a thematic approach to capture themes related to components of the theory of change and emergent themes in Nvivo (Ulin et al., 2005; Denzin & Lincoln, 2017). A. P., S. M. and M. P. constructed case studies by coding each individual interview using the coding framework reflecting the theory of change, then repeatedly reading interviews within each family set (e.g., daughter‐in‐law, mother‐in‐law and husband) to draft and revise narrative case study summaries drawing on each interview. Examples of these summaries are given in Prost et al. (2022). For this paper, A. P. reanalyzed the qualitative data with a focus on understanding the roles of intrahousehold power inequalities and cooperation in shaping household responses to the interventions, as well as individual, family and community factors that influenced this. To this end, A. P. did initial open coding of the data to capture emergent themes related to these categories, and then produced a coding framework that was refined iteratively in discussion with the wider study team, and systematically applied by A. P. to the data. Emergent themes were synthesized by A. P. and H. H‐F. to both explain quantitative findings and provide an alternative perspective on household cooperation (Creswell, 2014).

## 3 RESULTS

### 3.1 Sample characteristics

The respondent flow diagram is given in Supporting Information: Figure 2 and respondent characteristics are given in Supporting Information: Table 1. We had nonresponse rates of 17.4% at baseline and 10.5% at endline. Respondent characteristics are similar in all samples and arm‐wise summaries show respondent characteristics were well‐balanced (Kadiyala et al., 2021). Women and children consumed on average 3/7 and 4/10 food groups, respectively. Women and their husbands had on average 6 or 7 years of education, respectively. Women owned about a third of the household's assets and were involved (jointly or solely) in a mean of two out of seven decisions relating to agriculture or nutrition. Mothers typically worked longer hours than fathers (mean: 11 vs. 8 h/day) and spent more time on childcare (mean: 10 vs. 3 h/day).

### 3.2 Intrahousehold power inequalities, household cooperation and diets

Analyses of baseline data (Table 2) show that mothers owning a higher share of household assets had more diverse diets (adjusted mean difference: 0.5 food groups, SE: 0.09, *p* < 0.001). Although crude models find positive associations between women's assets and education shares and children's dietary diversity, associations are attenuated in adjusted models. We do not find associations between women's decision‐making and the dietary diversity of mothers or children. Household cooperation (men's care share) is not associated with mothers' diets but is positively associated with children's diets (adjusted mean difference 0.90 [SE 0.31], *p* = 0.004).

**Table 2:** Crude and adjusted associations between bargaining power or household cooperation and dietary intakes at baseline.

	Outcome: Mother dietary diversity score				Outcome: Child dietary diversity score			
*N*	*N*	Mean difference	(SE)	*p* Value	*N*	Mean difference	(SE)	*p* Value
Asset share								
Crude	4437	0.78	0.09	<0.001	3627	0.23	0.11	0.04
Adjusted	4377	0.50	0.09	<0.001	3593	0.05	0.10	0.60
Education share								
Crude	4436	0.31	0.08	<0.001	3627	0.33	0.10	0.001
Adjusted	4378	0.04	0.08	0.58	3595	0.15	0.09	0.10
Decision‐making								
Crude	4438	−0.02	0.01	0.10	3629	0.009	0.02	0.59
Adjusted	4378	−0.02	0.01	0.19	3595	−0.004	0.02	0.79
Men's care share								
Crude	1798	0.24	0.27	0.36	1469	1.50	0.34	<0.001
Adjusted^a^	1775	0.09	0.26	0.73	1455	0.90	0.31	0.004

*Note*: Men's care share measured in 50% of the sample. Child dietary diversity excludes children aged 0–6 months. Adjusted models control for caste, dependency ratio, land size, total household assets and total education, plus the mother's age for the mother's diet diversity outcome and the child's age for the child's dietary diversity outcome.

Abbreviation: SE, standard error.

^a^ Additional control: total care time.

Our qualitative analyses show that other forms of household cooperation play a role in shaping both women's and children's dietary diversity. Fathers‐in‐law and husbands described themselves as cooperative providers responsible for purchasing foods from nearby markets. In some families, fathers‐in‐law and husbands described cooperating to ensure that pregnant women and children would receive diverse diets.

P: Whatever my daughter‐in‐law asks for, I'll bring. […]

I: Were you bringing those things earlier or did you start bringing them now?

P: I brought them from the very beginning. […] When she was pregnant, whatever she needed, I brought for her.

(Father‐in‐law)

For many women, however, pregnancy increased dependency on male relatives who actively or passively ‘regulated’ their dietary diversity:

P: Previously he [husband] used to bring fish and meat once in every eight days but now it's once every fifteen days. […]

I: Why did you use to eat once in a week, and now once every fifteen days?

P: He doesn't bring it.

I: And what else do you eat other than meat and fish?

P: Rice and daal and… vegetables… if he brings them.

(Mother)

Other families faced limitations in buying or producing more food for women and children due to a lack of income and time:

I: Do you know anything about the food requirements of your wife and child?

P: I bring everything. I give them food.

I: Do you bring anything else? Seeing that you have a child now?

P: No.

I: Why don't you bring [anything]?

P: I bring as much as my money allows me.

(Husband)

Taken together, we find that households cooperate to produce and procure food for women and children, but some households are more responsive to changes due to pregnancy than others, and financial constraints also explain some heterogeneity.

### 3.3 A quantitative exploration of moderation and mediation of trial impacts

The UPAVAN interventions affected diets in different ways (Table 3). Compared with control, there were increases in mothers' mean dietary diversity score in AGRI‐NUT (0.13 food groups, *p* = 0.05) and AGRI‐NUT+PLA (0.23 food groups, *p* < 0.001), and perhaps AGRI (0.12 food groups, *p* = 0.08). There were also increases in children's mean dietary diversity in AGRI‐NUT+PLA (0.28 food groups, *p* < 0.001) but not AGRI or AGRI‐NUT. This is consistent with the main results for binary outcomes (Kadiyala et al., 2021).

**Table 3:** Effect of the UPAVAN interventions on diets, bargaining power and cooperation.

	Control	AGRI	AGRI‐NUT	AGRI‐NUT+PLA	AGRI vs. control			AGRI‐NUT vs. control			AGRI‐NUT+PLA vs. control		
Outcome	Mean (SD)	Mean (SD)	Mean (SD)	Mean (SD)	Mean difference	(SE)	*p* Value	Mean difference	(SE)	*p* Value	Mean difference	(SE)	*p* Value
Mother's dietary diversity (score out of 10)													
Baseline	*n* = 1058	*n* = 1106	*n* = 1091	*n* = 1183									
	3.8 (1.1)	3.6 (1.1)	3.7 (1.2)	3.8 (1.2)									
Endline	*n* = 991	*n* = 1093	*n* = 1047	*n* = 1129									
	4.1 (1.3)	4.1 (1.3)	4.2 (1.3)	4.3 (1.4)	0.12	0.07	0.08	0.13	0.07	0.05	0.23	0.07	<0.001
Children's dietary diversity (score out of 7)													
Baseline	*n* = 849	*n* = 917	*n* = 886	*n* = 977									
	2.8 (1.3)	2.8 (1.3)	2.7 (1.3)	2.7 (1.3)									
Endline	*n* = 751	*n* = 817	*n* = 805	*n* = 854									
	3.1 (1.3)	3.1 (1.3)	3.2 (1.4)	3.4 (1.3)	−0.002	0.07	0.98	0.12	0.07	0.11	0.28	0.07	<0.001
Women's asset share^a^													
Baseline	*n* = 1057	*n* = 1107	*n* = 1091	*n* = 1184									
	0.3 (0.2)	0.3 (0.2)	0.3 (0.2)	0.4 (0.2)									
Endline	*n* = 991	*n* = 1093	*n* = 1047	*n* = 1129									
	0.3 (0.1)	0.3 (0.1)	0.3 (0.1)	0.3 (0.1)	−0.04	0.009	<0.001	−0.0008	0.01	0.94	−0.06	0.009	<0.001
Women's education share^b^													
Baseline	*n* = 1059	*n* = 1107	*n* = 1094	*n* = 1183									
	0.4 (0.2)	0.4 (0.2)	0.4 (0.2)	0.4 (0.2)									
Endline	*n* = 990	*n* = 1092	*n* = 1047	*n* = 1128									
	0.5 (0.2)	0.4 (0.2)	0.5 (0.2)	0.4 (0.2)	−0.001	0.01	0.92	0.01	0.01	0.36	−0.008	0.01	0.46
Women's decision‐making													
Baseline	*n* = 1060	*n* = 1108	*n* = 1094	*n* = 1184									
	2.2 (1.3)	2.1 (1.2)	2.2 (1.3)	2.1 (1.3)									
Endline	*n* = 991	*n* = 1093	*n* = 1047	*n* = 1129									
	2.7 (1.6)	3.0 (1.5)	3.0 (1.6)	2.8 (1.5)	0.39	0.08	<0.001	0.27	0.08	<0.001	0.13	0.08	0.08
Men's care share^c^													
Baseline	*n* = 436	*n* = 447	*n* = 439	*n* = 476									
	0.24 (0.10)	0.26 (0.11)	0.24 (0.09)	0.25 (0.10)									
Endline	*n* = 318	*n* = 361	*n* = 328	*n* = 389									
	0.27 (0.13)	0.27 (0.12)	0.27 (0.14)	0.27 (0.12)	−0.009	0.009	0.34	−0.002	0.01	0.83	−0.01	0.009	0.25

*Note*: Men's care share only measured in 50% of the sample. Child dietary diversity excludes children aged 0–6 months. All models adjust for baseline measures of the outcome.

Abbreviations: SD, standard deviation; SE, standard error; UPAVAN, Upscaling Participatory Action and Videos for Agriculture and Nutrition.

^a^ Additional control: total household assets.

^b^ Additional control: total education.

^c^ Additional control: total care time.

As hypothesized, results in Table 3 show no differences in education share and care share between intervention arms and control. However, women do have lower shares of assets in AGRI and AGRI‐NUT+PLA arms. We, therefore, do not explore moderation by women's asset share. Moderation of intervention effects on diets by high versus low levels of relative power (women's education share) or cooperation (men's care share) is reported in Table 4, showing no clear evidence that the effects of the interventions varied by these indicators of power inequalities or household cooperation.

**Table 4:** Moderation of the effect of UPAVAN interventions on dietary diversity by bargaining power or household cooperation.

			AGRI vs. control		AGRI‐NUT vs. control		AGRI‐NUT+PLA vs. control		
Outcome	Moderator variable	Moderator level	Mean diff by moderator level	(SE)	Mean diff by moderator level	(SE)	Mean diff by moderator level	(SE)	Interaction *p* value
Mother's dietary diversity score	Women's education share^a^	Low	0.19	0.08	0.12	0.08	0.17	0.08	0.13
		High	0.09	0.07	0.17	0.07	0.30	0.07	
	Men's care share^b^	Low	0.14	0.13	−0.08	0.13	0.13	0.13	0.06
		High	−0.02	0.12	0.24	0.12	0.19	0.12	
Children's dietary diversity score	Women's education share^a^	Low	0.06	0.09	0.16	0.09	0.23	0.09	0.70
		High	0.01	0.08	0.11	0.08	0.31	0.08	
	Men's care share^b^	Low	0.07	0.15	0.13	0.16	0.23	0.15	0.50
		High	−0.17	0.14	0.19	0.15	0.22	0.14	

*Note*: Moderation by women's asset share was not reported because of differences between arms shown in Table 3. Men's care share was only measured in 50% of the sample. Child dietary diversity excludes children aged 0–6 months. All models adjust for baseline measures of the outcome.

Abbreviations: PLA, participatory learning and action; SE, standard error; UPAVAN, Upscaling Participatory Action and Videos for Agriculture and Nutrition.

^a^ Additional control: total education.

^b^ Additional control: total care time.

Next, we consider whether changes in decision‐making power could be mediating effects of UPAVAN interventions. We do find evidence that UPAVAN interventions affected both mediators and outcomes (Table 3). The number of decisions women made was slightly higher in AGRI, AGRI‐NUT and possibly AGRI‐NUT+PLA arms, compared with the control, and we have already shown that dietary diversity was higher in interventions compared with the control. However, when we control for intervention, decision‐making does not affect maternal dietary diversity (mean difference: −0.02, SE: 0.009, *p* = 0.10), or children's dietary diversity (mean difference: 0.003, SE: 0.01, *p* = 0.80). Therefore, we do not find quantitative evidence that the effects of the interventions on diets were mediated by increases in women's decision‐making.

### 3.4 Qualitative exploration of moderation and mediation of trial impacts

Our qualitative data suggest that unmeasured dimensions of power inequalities and cooperation matter, including the confidence to speak up and interpersonal relationships within households.

#### 3.4.1 Power and cooperation to increase the consumption of foods available

Viewing and discussing UPAVAN nutrition‐specific videos gave women information about the importance of diverse diets, and motivation and confidence to ask for specific foods for themselves or their children.

I: [Echoing the participant] After watching the video, you started talking about food and eating well in your house. Did you used to speak like that before?

P: No, I couldn't have said these things before, but we were eating these things.

I: Now that you are saying these things, what do you feel? …

P: I am feeling good.

(Mother, AGRI‐NUT)

Agreeing with interviews from parents‐in‐law, daughters‐in‐law reported that their decision‐making power concerning diets was increased by going to meetings, gaining knowledge and because frontline workers' advice echoed what they said. In addition, the fact that many meetings included both daughters‐ and mothers‐in‐law meant that their understanding of nutrition converged. Even mothers‐in‐law who did not come to meetings felt pressure to comply with the ‘modern’ advice offered to women by videos and frontline workers. One mother‐in‐law said:

P: We tell them not to eat spinach and ridge gourd. But they say ‘we have seen [it] in the video, so we may not eat it regularly, but we can eat it sometimes’.

I: Don't you stop them? [from eating these foods]

P: What will happen if we try to stop them?

(Mother‐in‐law of pregnant woman, AGRI‐NUT+PLA)

Several group facilitators across the trial arms also actively negotiated improvements in diets with family members:

One mother said ‘I will not eat poi [malabar spinach] leaves because my mother‐in‐law said no’. […] Then in the evening, I went to her house and made her mother‐in‐law understand the benefits. ‘Don't tell your daughter‐in‐law this. In today's world, nutritious things must be consumed’.

(UPAVAN facilitator, AGRI‐NUT)

Women gained additional benefits from the PLA component in the AGRI‐NUT+PLA intervention. As part of the meeting cycle, group members developed strategies to address barriers to accessing diverse foods. Importantly, the fact that women were given a chance to speak up in meetings gave them the confidence to speak up in other contexts, including at home.

#### 3.4.2 Power and cooperation to increase household production of food

Qualitative data showed that changes to agricultural practices linked to the UPAVAN interventions were heterogeneous, and conditional on household cooperation. Women usually told their families about the content of the NSA videos. Some did so to show that they were learning useful things and that it was worth going to meetings but did not start conversations about taking up new practices or changing existing ones, often because of constraints such as lack of land, water or support from husbands, and fathers‐ or mothers‐in‐law. Husbands and fathers‐in‐law migrating for work placed limitations on household cooperation and labour inputs to support cultivation, as they were away for prolonged periods. In the example below, a mother saw videos but did not adopt new agricultural practices because she felt their land was too small and her husband would give limited support:

P: After I watched videos, they [family] asked me, what did you see? So I told them. […]

I: Did you use the same methods in both years [before and after watching videos]?

P: Yes, because of shortage of space and because my husband has very limited time…

(Mother, AGRI arm)

Many women discussed the content of videos and meetings to enlist support from their husbands, fathers‐in‐law or mothers‐in‐law to start new, small‐scale cultivation in their homestead garden by themselves, or with minimal support from others. A key supporting factor for this was having husbands, fathers‐in‐law or mothers‐in‐law who were already interested and engaged in cultivation, whether on a small or large scale:

I: Have you talked with your family about how to stay healthy and how to grow vegetables?

P: Yes. We'll benefit from farming. We definitely talk about this. Our entire work is farming.

I: Who did you discuss the videos with?

P: My husband, mother‐in‐law… My father in‐law also does cultivation. […].

(Pregnant woman, AGRI‐NUT)

In households that made profits by changing their cultivation practices due to videos, women felt that their status had increased because they were able to contribute to agricultural decisions, household health and economic well‐being. Cultivation successes could also increase women's control over household expenditures:

P: Now after seeing the video we know many things. We are also sharing these in front of our family members […]. I feel happy about this because my family members respect what I say.

I: Were you able to tell your family members to spend money in certain ways before?

P: No, earlier I did not say anything because what was cultivated was enough for our use. There was no surplus. So I did not say anything about this. But now we are harvesting more, so we can sell the remaining vegetables. And I can tell my family member to keep that money as our savings because in the future we can use those for emergencies, or if we need anything to buy seeds and household items, then we can buy those using this money. I can tell these things to my family members now.

(Mother, AGRI)

In all cases, however, the extent to which women suggested taking up new practices or changing existing ones depended on the degree of cooperation they anticipated from family members. In practice, decisions about cultivation had to involve most members, to attain their active participation or assent.

Some women lived in households in which cultivation and spending decisions were largely controlled by fathers‐ or mothers‐in‐law, and were afraid to speak up. This was especially the case for younger, newly married women:

I: Who cultivated in your family?

P: Father.

I: Father‐in‐law does. Did you talk with him and make suggestions?

P: No.

I: Did you discuss [anything] after watching that video?

P: No.

I: Why didn't you discuss?

P: I fear him (in a laughing voice). I fear my father‐in‐law.

I: What else?

P: Nothing else.

(Mother, AGRI‐NUT)

Other women lived with more supportive families that cooperated mainly by ‘allowing’ women to add a few more new crops to the homestead garden, buying seeds and occasionally helping with the planting.

Land ownership also affected cooperation and control over decisions. In most cases, the land was owned by fathers‐in‐law or husbands, and families worked together to produce crops. Families listened to the daughter‐in‐law's suggestions, but daughters‐in‐law also knew that they should do as they were told because they worked on their fathers‐in‐law's land:

P: What will the daughter‐in‐law do? As our lands are together, our cultivation will definitely be done together, isn't it? […]

I: If she would wish something else because she is going to watch videos, if she wished to do some other kind of agriculture and she told you about that, then would you follow her advice? […]

P: They're not living [here] as our daughters‐in‐law, they're living as our daughters. Whatever we'll say, they'll move in that way.

(Mother‐in‐law, AGRI‐NUT‐PLA)

Decisions about selling crops were also often taken by husbands and fathers‐in‐law, with daughters‐in‐law having a say in whether to keep some crops for consumption but not the timing of sales or pricing:

I: You said that you sell potato, eggplant, and tomato. Do you control anything about the sale of these vegetables?

P: No.

I: You don't control anything. Don't you have a say in any decision?

P: No, he takes the vegetable and sells them.

I: Do you say anything about the sale?

P: Yes, he always asks ‘should we eat or sell'? I suggest to him that we can eat some and sell the rest.

(Pregnant woman, AGRI)

In summary, although quantitative analyses showed no evidence of effect modification by indicators of power inequalities and household cooperation, qualitative evidence indicates that interventions were more likely to lead to changes in agricultural practices in more supportive, cooperative households. In some cases, the interventions increased women's decision‐making power, consistent with the quantitative evidence showing some increases in women's decision‐making. However, women's decisions to enter into negotiations with other family members depended on whether women deemed the practices to be feasible, women's confidence, and previous cultivation success. This heterogeneity may explain why we do not find evidence that decision‐making mediated the effects of the interventions on diet quality.

## 4 DISCUSSION

We have shown that household cooperation and women's relative power in rural Odisha may influence children's and mothers' dietary diversity, and partly determine the effects of NSA interventions tested in UPAVAN. The qualitative evidence shows that interventions were more effective in cooperative families—especially when other conditions were met, such as having access to land, water and labour. They also increased women's decision‐making around food and agriculture, particularly among households that had resources to act upon new nutrition or agriculture knowledge. This variation in the extent to which cooperation and power inequalities affect the adoption of UPAVAN‐promoted practices may explain why we do not find quantitative evidence of mediation or moderation.

Our qualitative results indicate that NSA interventions may be more effective if they include multiple family members. In UPAVAN, the NSA interventions engaged family members in various ways. Mothers‐in‐law participated in meetings; participants were encouraged to discuss the intervention at home; and facilitators visited participants' homes, allowing interaction with other members. To further enhance the inclusion of other family members, additional intervention components could be integrated, while ensuring that the ‘women's space' created by SHGs is not undermined. This could involve integrating home‐based counselling interventions that facilitate intra‐household dialogue and joint decision‐making (Morrison et al., 2021), and mixed groups involving mothers and fathers, such as parenting or antenatal classes (Brixval et al., 2015), or nutrition education involving mothers, fathers and grandmothers (Thuita et al., 2021).

This is consistent with a growing body of work showing the importance of grandmothers (Concha & Jovchelovitch, 2021), other women (Usman et al., 2021) and spouses (Morrison et al., 2021) in shaping maternal and child nutrition‐related behaviours, and in increasing the impact of behaviour change interventions (Narayanan & Rao, 2019; Thuita et al., 2021). In line with this, nutrition interventions that engage other families are growing in popularity—a systematic review identified 67 studies that include the wider family in some way (Martin et al., 2021).

Household engagement may be even more important in NSA interventions because men typically have more control over agricultural than nutritional decisions, because women face other gender‐specific barriers in adopting new agricultural practices (Jewitt, 2000), and because agricultural interventions may increase women's workload (Jewitt, 2000). UPAVAN deliberately promoted practices that were time‐saving or required low labour, but more work to increase households' willingness to share the responsibility of adopting new practices may be required to increase impact.

Both quantitative and qualitative results also indicate that UPAVAN interventions increased women's decision‐making power, and qualitative evidence shows that this aided their adoption of NSA‐promoted practices in some cases but not all. This may be because the UPAVAN interventions provided space for women to gain confidence and practice speaking up, but land, labour and water constraints remained insurmountable for some. Other studies from Eastern India support the conclusion that women's power determines a household's propensity to adopt new livelihood strategies (Savath et al., 2014).

These findings are highly relevant to the large ongoing NSA programmes in eastern India, including Odisha's ‘nutri‐garden’ project in 30 districts (Government of India, 2020), and a multi‐sectoral intervention that includes homestead cultivation (*Swambhimaan*) in three states (Sethi et al., 2019). These programmes partly rely on SHGs to approach women, but the involvement of other family members is not described in detail. Our analyses raise important questions for these programmes: Do they mainly influence diets by increasing women's influence over homestead gardens? What role do prior (preintervention) and intervention‐generated power balances and cooperation play in moderating or mediating impacts?

Our study has some limitations: Intrahousehold power inequalities and cooperation are difficult to measure quantitatively; work is especially needed to develop indicators of household cooperation. For the qualitative exploration of intrahousehold power inequalities and cooperation, we did a secondary analysis of qualitative data collected as part of UPAVAN's overall process evaluation; focusing data collection specifically on exploring household cooperation and power inequalities might have yielded richer data more amenable to triangulation. Heterogeneity in household dynamics means that we are unable to explore every source comprehensively; future research could focus on identifying which sources of heterogeneity matter most. With our study design, we cannot infer causal effects of intrahousehold power inequalities or household cooperation on diets. Finally, social desirability bias may encourage respondents to be overly positive about the intervention. However, interviewers were hired by DCOR (who were not involved in the intervention) and trained to build rapport with participants. The wide range of barriers that respondents shared suggests they felt comfortable speaking freely.

We conclude that NSA interventions may be more effective if they can find ways to elicit full cooperation from the whole household. Interventions would benefit from incorporating the role of household cooperation and power inequalities in their intervention design and theories of change. There is also wide heterogeneity in intrahousehold dynamics and the constraints that families face. Further research is needed to identify how interventions can respond to this heterogeneity, by alleviating resource and labour constraints where possible and co‐designing socially acceptable ways to increase cooperation and reduce power inequalities. With this information, interventions could be designed so that all families can be engaged in an inclusive and empowering way.

## AUTHOR CONTRIBUTIONS

Helen Harris‐Fry conceived the research study, with inputs from Suneetha Kadiyala, Audrey Prost, Satyanarayan Mohanty and Manoj Parida. Satyanarayan Mohanty and Manoj Parida led data collection. Emily Fivian and Emma Beaumont led the quantitative analyses, with inputs from Helen Harris‐Fry, Suneetha Kadiyala and Elizabeth Allen. Audrey Prost led the qualitative analysis, with inputs from Helen Harris‐Fry and Suneetha Kadiyala. Suneetha Kadiyala, Elizabeth Allen, Audrey Prost and Helen Harris‐Fry oversaw the conduct of the trial. Shibanath Padhan and Naba K. Mishra implemented the interventions, with coordination from Ronali Pradhan and Satyapriya Sahu, with technical inputs from Peggy Koniz‐Booher, Shibanand Rath and Suchitra Rath. Helen Harris‐Fry and Audrey Prost wrote the first draft of the manuscript, with inputs from all co‐authors.

## CONFLICT OF INTEREST STATEMENT

The authors declare no conflict of interest.

## ETHICAL STATEMENT

Ethics approval was obtained from the Odisha government's Institutional Review Board (Reference: 141/SHRMU) and LSHTM Interventions Research Ethics Committee (Reference: 11357). Trial registration: ISRCTN65922679. We sought written consent for participation in interviews or discussions, audio recording and the use of pseudonymized data from participants.

## Supporting information

Supporting information.

## Data Availability

Data are available on request from the corresponding author.
