# Supplementary material for: Intrahousehold power inequalities and cooperation: Unpacking household responses to nutrition‐sensitive agriculture interventions in rural India
Source: Matern Child Nutr. 2023 Mar 20;19(3):e13503. doi: 10.1111/mcn.13503 (PMC10262904; doi:10.1111/mcn.13503)
Supplement: Supplementary file 1 — Supporting information. [file MCN-19-e13503-s001.docx]

Supplemental Materials

## Supplemental Figure 1 UPAVAN theory of change

**Supplemental Figure 2: Respondent flow diagram**


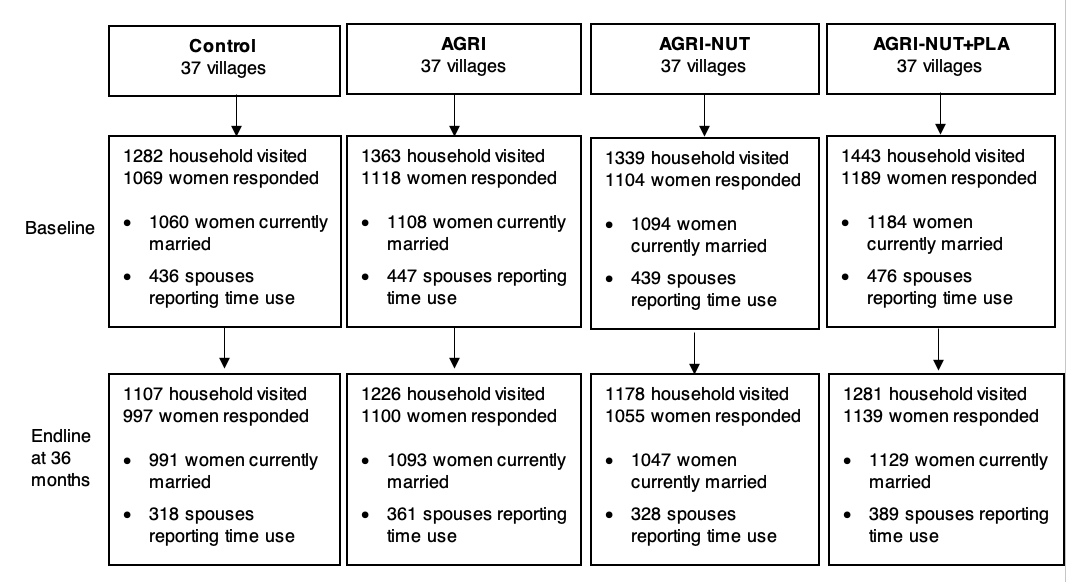


**Supplemental Table 1: Respondent characteristics**

| **Characteristic** | **Baseline**  **Full sample**  ***N*=4446** | | **Baseline**  **50% sub-sample**  ***N*=1798** | | **Baseline + Endline**  **Full sample**  ***N*=8706** | | **Baseline + Endline, 50% sub-sample**  ***N*=3194** | |
| --- | --- | --- | --- | --- | --- | --- | --- | --- |
| **Sociodemographic characteristics** | ***N*** | **Mean (SD) or *n* (%)** | ***N*** | **Mean (SD) or *n* (%)** | ***N*** | **Mean (SD) or *n* (%)** | ***N*** | **Mean (SD) or *n* (%)** |
| Mother’s age in years, mean (sd) | 4435 | 24.5 (4.0) | 1794 | 24.5 (4.1) | 8695 | 24.6 (4.2) | 3190 | 24.7 (4.2) |
| Father’s age in years, mean (sd) | 4439 | 33.9 (12.0) | 1798 | 30.3 (6.2) | 8695 | 34.3 (12.3) | 3194 | 30.3 (6.0) |
| Child’s age in months, mean (sd) | 4445 | 12.3 (6.5) | 1798 | 12.2 (6.4) | 8705 | 12.1 (6.6) | 3194 | 12.1 (6.6) |
| Number of adults (≥10 years), mean (sd) | 4445 | 3.6 (1.8) | 1798 | 3.5 (1.6) | 8705 | 3.5 (1.7) | 3194 | 3.4 (1.5) |
| Number of children (<10 years), mean (sd) | 4445 | 1.8 (0.9) | 1798 | 1.8 (0.9) | 8705 | 1.8 (0.9) | 3194 | 1.8 (0.9) |
| Size of landholdings, n (%) | 4400 |  | 1782 |  | 8645 |  | 3174 |  |
| <2.5 acres |  | 3558 (80.9) |  | 1468 (82.3) |  | 6973 (80.7) |  | 2607 (82.1) |
| ≥2.5 aces |  | 842 (17.8) |  | 316 (17.7) |  | 1672 (19.3) |  | 567 (17.9) |
| Caste, n (%) | 4439 |  | 1798 |  | 8695 |  | 3194 |  |
| Scheduled Caste |  | 402 (9.1) |  | 157 (8.7) |  | 773 (8.9) |  | 280 (8.7) |
| Scheduled Tribe |  | 2594 (58.4) |  | 1076 (59.8) |  | 5128 (59.0) |  | 1906 (59.7) |
| Other Backward Castes & Other Castes |  | 1443 (32.5) |  | 565 (31.4) |  | 2794 (32.1) |  | 1008 (31.6) |
| **Bargaining power** |  |  |  |  |  |  |  |  |
| Woman’s asset count, mean (sd) | 4441 | 2.0 (1.4) | 1798 | 1.9 (1.3) | 8701 | 2.0 (1.2) | 3194 | 1.9 (1.2) |
| Household asset count, mean (sd) | 4442 | 6.0 (1.6) | 1797 | 5.9 (1.6) | 8702 | 6.0 (1.6) | 3193 | 5.9 (1.6) |
| Woman’s asset share, mean (sd) | 4439 | 0.3 (0.2) | 1797 | 0.3 (0.2) | 8699 | 0.3 (0.2) | 3193 | 0.3 (0.2) |
| Woman’s education in years, mean (sd) | 4445 | 6.4 (4.5) | 1798 | 6.2 (4.4) | 8705 | 6.6 (4.5) | 3194 | 6.4 (4.5) |
| Spouse’s education in years, mean (sd) | 4443 | 7.3 (4.2) | 1797 | 7.1 (4.2) | 8700 | 7.4 (4.1) | 3193 | 7.2 (4.1) |
| Woman’s education share, mean (sd) | 4443 | 0.4 (0.2) | 1798 | 0.4 (0.2) | 8700 | 0.4 (0.2) | 3194 | 0.4 (0.2) |
| Women’s decision-making, mean (sd) | 4446 | 2.2 (1.3) | 1798 | 2.2 (1.2) | 8706 | 2.5 (1.4) | 3194 | 2.5 (1.4) |
| **Cooperation and time use** |  |  |  |  |  |  |  |  |
| Mother’s hours on childcare, mean (sd) | - | - | 1798 | 9.8 (2.6) | - | - | 3194 | 9.6 (2.9) |
| Father’s hours on childcare, mean (sd) | - | - | 1798 | 3.3 (1.8) | - | - | 3194 | 3.4 (2.0) |
| Men’s care share | - | - | 1798 | 0.2 (0.1) | - | - | 3194 | 0.3 (0.1) |
| Mother hours on other work, mean (sd) | - | - | 1798 | 11.0 (2.4) | - | - | 3194 | 11.0 (2.4) |
| Father hours on other work, mean (sd) | - | - | 1798 | 8.1 (3.0) | - |  | 3194 | 8.6 (3.0) |
| **Dietary diversity** |  |  |  |  |  |  |  |  |
| Women’s dietary diversity score, mean (sd) | 4438 | 3.7 (1.1) | 1798 | 3.7 (1.1) | 8698 | 3.9 (1.3) | 3194 | 3.9 (1.2) |
| Number of children ≥ 6 months | 3637 |  | 1471 |  | 6985 |  | 2568 |  |
| Child’s dietary diversity score, mean (sd) | 3629 | 2.8 (1.3) | 1469 | 2.7 (1.3) | 6856 | 3.0 (1.3) | 2520 | 3.0 (1.3) |
